# Quantification of lithium at ppm level in geological samples using nuclear reaction analysis

**DOI:** 10.1007/s10967-018-5907-0

**Published:** 2018-05-28

**Authors:** Nathaly De La Rosa, Per Kristiansson, E. J. Charlotta Nilsson, Linus Ros, Jan Pallon, Henrik Skogby

**Affiliations:** 1 Division of Nuclear Physics, Department of Physics, Lund University, Box 118, 221 00 Lund, Sweden; 2 Department of Geosciences, Swedish Museum of Natural History, Box 50007, 104 05 Stockholm, Sweden

**Keywords:** Nuclear reaction analysis (NRA), Lithium quantification, Particle induced X-ray emission (PIXE), Geological samples, Double-sided silicon strip detector (DSSSD)

## Abstract

Proton-induced reaction (p,α) is one type of nuclear reaction analysis (NRA) suitable especially for light element quantification. In the case of lithium quantification presented in this work, accelerated protons with an energy about of 850 keV were used to induce the ^7^Li(p,α)^4^He reaction in standard reference and geological samples such as tourmaline and other Li-minerals. It is shown that this technique for lithium quantification allowed for measurement of concentrations down below one ppm. The possibility to relate the lithium content with the boron content in a single analysis was also demonstrated using tourmaline samples, both in absolute concentration and in lateral distribution. In addition, Particle induced X-ray emission (PIXE) was utilized as a complementary IBA technique for simultaneous mapping of elements heavier than sodium.

## Introduction

Lithium, as an alkali metal, is a highly reactive element, and as a consequence of this, lithium does not appear as an isolated metal in nature. Instead, it combines with other elements to form minerals or other natural compounds [1]; for instance, lithium occurrence is common in some ferromagnesian minerals where it partly substitutes magnesium [2].

Lithium can be found in many natural brines, nearly all igneous rocks, as a low concentration constituent. Lithium is also found as a stoichiometric component in some minerals such as spodumene, lepidolite, petalite, and amblygonite [3]. J. Arfwedson discovered lithium as an elemental species in petalite at the beginning of the 19th century but industrial relevance came around forty years later [2]. Nowadays, lithium is of great interest to investigate for instance, the primordial lithium problem in cosmology [4], treatment of bipolar disorder, Alzheimer’s disease, and thyroid cancer [5], as shielding material in nuclear reactors [6], in Li-ion batteries [7] and in geological processes such as magmatic differentiation, seawater-basalt interaction [8] and crust-mantle recycling [9].

In many of the aforementioned applications, techniques capable of measuring as low concentrations of lithium as a few ppm are required. Ion induced reactions, elastic recoil detection analysis (ERDA), and neutron induced methods are sensitive nuclear techniques that have been utilized to quantify lithium [10]. Proton induced reactions are one type of ion induced reactions most commonly chosen for analysis of lithium. Proton irradiation can yield prompt γ-ray emission, neutron emission, α-particle emission, etc., depending mainly on the energy of the incident proton [11].

In the study reported in this paper, lithium analysis in geological samples was carried out at the Lund Ion Beam Analysis Facility (LIBAF) using the reaction ^7^Li(p,α)^4^He. This reaction has been selected for lithium quantification, since it has a high Q-value of about 17 MeV, a favorable cross-section [12] and produces two α particles that are easily distinguished from scattered beam particles and other reaction products [13]. The ^7^Li(p,α)^4^He cross-section increases smoothly in the proton beam energy interval 0–2.5 MeV with a maximum of 3.3 mb/sr at 2.5 MeV. In the present work, the lithium reaction was induced by protons with energies of 852 keV. At this energy, the cross-section is 0.5 mb/sr at 150° [12]. Particle Induced X-ray Emission (PIXE) was performed simultaneously with Nuclear Reaction Analysis (NRA) and hence enabled the possibility to study spatial correlations between concentrations of heavier matrix elements and the lithium.

In previous work [14], the reaction ^7^Li(p,α)^4^He yield and detection limits were studied as a function of the energy of the incident proton beam. In that work, we confirmed that the NRA technique can be routinely used for measuring lithium in geological materials, with concentrations down to a few ppm.

In order to further illustrate the capability of the technique and contribute to a number of geological investigations (e.g. [15],) selected geological samples belonging to the tourmaline group were analyzed. Tourmaline is a borosilicate mineral that presents a constant concentration of boron (approx. 3%) with a quite variant composition [16]. Its generic formula is

1 $${\text{XY}}_{3} {\text{Z}}_{6} \left( {{\text{T}}_{6} {\text{O}}_{18} } \right)\left( {{\text{BO}}_{3} } \right)_{3} {\text{V}}_{3} {\text{W}},$$

where the X, Y, Z, T, V and W sites can be occupied as following, X = Na, Ca, K, or vacancies; Y = Mg, Fe, Mn, Li, Cr, Al; Z = Al, Mg, Ti, V, Cr, Fe; T = Si, Al; V = OH, O; W = OH, F, O. Tourmalines are organized according to the type of element that occupies the X, Y, Z, T, V and W sites [17]. For the alkali-group tourmalines, elbaite is the lithium-bearing type, Na(Li, Al)_3_Al_6_(Si_6_O_18_)(BO_3_)_3_(OH,F)_4_, dravite is the magnesian, NaMg_3_ Al_6_(Si_6_O_18_)(BO_3_)_3_(OH,F)_4_, and schorl the ferrous type Na(Fe,Mn)_3_ Al_6_(Si_6_O_18_)(BO_3_)_3_(OH,F)_4_ [18].

Tourmaline group minerals are complex borosilicates and their crystal structure and crystal chemistry have been widely studied. They are known as valuable indicator minerals that can provide information on the compositional evolution of their host rocks, chiefly due to their ability to incorporate a large number of elements. They frequently show intricate crystal-growth patterns related to changes in the crystallization environment [19].

## Experimental

Probing protons, capable of triggering the nuclear reaction ^7^Li(p,α)^4^He, were generated with the single-ended 3MV Pelletron accelerator located at LIBAF. Detailed descriptions of the LIBAF are published in Refs. [20, 21]. The samples were irradiated with a focused 3 nA proton beam with a 50 μm diameter. The proton beam energy was selected, based on the previous work [14], to be 852 keV, since at this energy the peaks from the reactions ^7^Li(p,α)^4^He, ^19^F(p,α)^16^O, and ^11^B(p,α)2α will be well-separated in the energy spectrum. The choice of beam energy took also into account factors such as lithium cross section and interferences from other reactions.

**Fig. 1:**
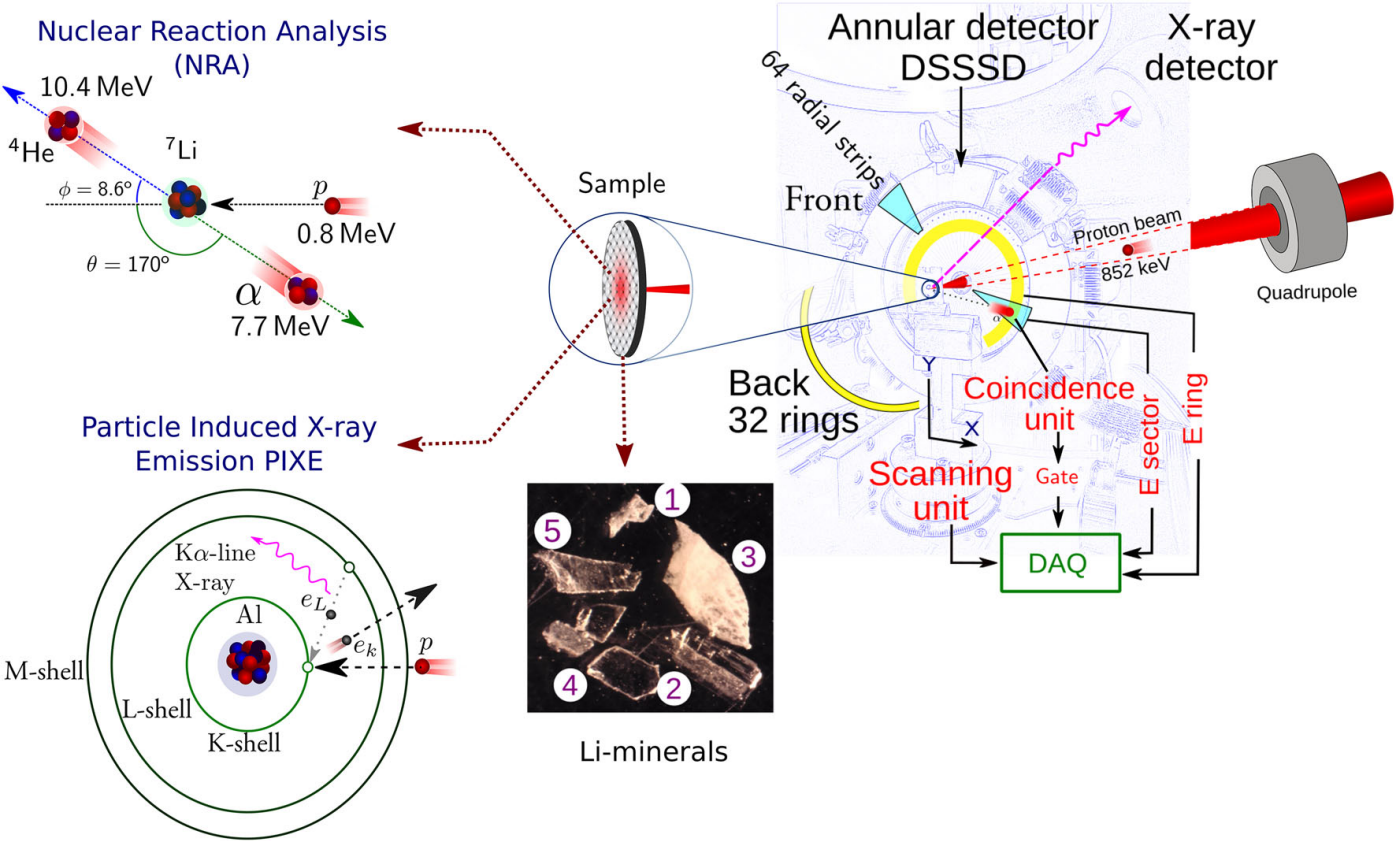
Schematic illustration of the experimental set-up. Energetic protons can induce α-particle and X-ray emissions in materials. In NRA, a proton at 0.8 MeV reacts with a target ^7^Li nucleus producing two α-particles. One is ejected in the backward direction relative to the beam and the second in the forward direction. The reaction parameters shown are calculated based on kinematic equations. Only α-particles ejected in the backward direction were detected with an annular detector. When an α-particle hits the DSSSD two energy signals are generated, one from a sector (front side) and other from a ring (back side). The simultaneous detection of those signals produces a gate that is processed by the DAQ system. In PIXE, inner shell ionizations caused by the proton beam are followed by electron deexcitations to fill inner shell vacancies and characteristic X-ray emission. The samples were scanned with a proton beam at 852 keV. The photo shows one of the samples used; it has five Li-minerals whose names are listed in Table 1

In Fig. 1, the experimental arrangement is schematically illustrated. The α-particles produced in the reaction and emitted in backward direction, were detected using a double-sided silicon strip detector (DSSSD) with 2048 pixels. This detector is a hollow disk with a thickness of 310 μm. Its outer and inner active diameters are 85 mm and 14 mm respectively, which provides an active solid angle of 2 sr, assuming a sample located 26 mm from the detector. In total, there are 96 strips, 64 stripes on front and 32 concentric rings on the back, each operated as a single surface barrier detector with e.g. similar shaping time and count rate behavior. A thorough explanation and technical specifications of the DSSSD can be found in Ref. [22].

The fact that the DSSSD is pixelated enables the use of high beam currents for NRA experiments, as the pixilation gives excellent pile-up suppression capability by the possibility to set multiplicity conditions on an event-by-event basis. With a traditional annular detector covering approximately the same solid angle a beam current around 200 pA was the typical possible to use. With the DSSSD, dividing the particle flux between many pixels a typical current convenient to use is instead 5 nA. With this current it is still possible to focus the beam to get a spatial resolution better than 10 μm. In the present system the limiting factor is the data rate that can be transferred and stored, but for the type of reactions studied here this can be solved by increasing the threshold.

Simultaneously with the NRA, PIXE analysis was carried out, as this technique enables the study of the presence of heavier elements in the samples, thus, it is possible to study correlations between heavier elements and Li. A 50 mm^2^ silicon drift detector was employed to identify the characteristic X-rays [23].

Samples of well-known chemical composition, as minerals with stoichiometric Li contents and standard reference materials, and a set of tourmaline crystals were utilized in the experiment. Lithiophosphate, amblygonite, spodumene, petalite and eucryptite were the Li-minerals employed, whose lithium concentration ranges from 2 to 18%. Two Standard Reference Materials (SRM) produced by the National Institute of Standards and Technology (NIST) were also used. The standard reference samples are SRM 610 and SRM 612 and they have a lithium concentration of 493(20) ppm and 44(3) ppm respectively [24, 25]. In addition, eight tourmaline samples were analyzed in order to study the relation between boron and lithium concentration and a Li-free synthetic quartz (Suprasil quartz crystal) [26] was analyzed to estimate the background, i.e. the signal under the Li-peak.

According to the reaction under study, lithium presence in samples is associated with detection of α-particles emitted in the reaction. Therefore, Li-mineral samples with a known composition were used to investigate the relation between lithium concentration δ_Li_ and the number of detected α-particles. Lithium concentration δ_Li_ is the lithium percentage calculated based on stoichiometric formulas, and the number of α-particles is the integrated peak counts (C_Li_) in the energy range of 7–8.1 MeV in an energy spectrum.

In order to compare the C_Li_ values from various samples, C_Li_ values are extracted from α-particle spectra, that should be normalized to the total charge used for the irradiation and compensated for the live time of the DAQ system, resulting in the Li yield from the sample, Y_Li_ = C_Li_/Q. Details about this normalization process are given in Ref. [27]. Q is the total number of impinging proton on the sample, which is measured with a precision of 3% using a pre-chamber Faraday cup and a fast-blanking electrostatic beam deflector [28]. To achieve C_Li_ values comparable between different samples, there is also a need to normalize C_Li_ to the volume analyzed since the range of the beam particles depends of the matrix composition. The reason is that the energy loss per length unit depends mainly on the electron concentration and this is related to the ratio between protons and neutrons in the matrix nuclei. For samples with a major element composition with the p/n ratio around one, the variations of the correction between different matrixes are in the order of a few percent, but e.g. samples in the rare earth element (REE) region requires much larger volume corrections.

The ranges R_i_ of different samples were calculated using the Stopping and Range of Ions in Matter software (SRIM) [29]. Thus, the factor R_SP_ for each sample is the ratio of its range R_i_ and the range in the reference sample, R_H_. This correction was applied only to samples with a well-known chemical composition such as the Li-minerals. For unknown or partly known samples an additional error should be added.

Plotting Y_Li_ as a function δ_Li_ render an expression of δ_Li_ for any Y_Li_, hence it was possible to quantify the Li content in samples that have an unknown concentration of lithium, here illustrated by a set of tourmalines. These samples have a high boron content, which was possible to measure simultaneously, since the reaction ^11^B(p,α)2α can occur under the same conditions as used for lithium analysis. The energy distribution of the α-particles produced in proton-^11^B reactions is extended to 6 MeV. According to Ref. [27], boron analysis can best be performed using a narrow energy interval 3.3–4.3 MeV. In order to assess the relation between boron concentration δ_B_ and boron yield Y_B_, the NIST samples and a tourmaline crystal were analyzed according to the method described in Ref. [27]. Thus, it was possible to study lithium content as a function of boron content in tourmaline samples.

## Results and discussion

**Fig. 2:**
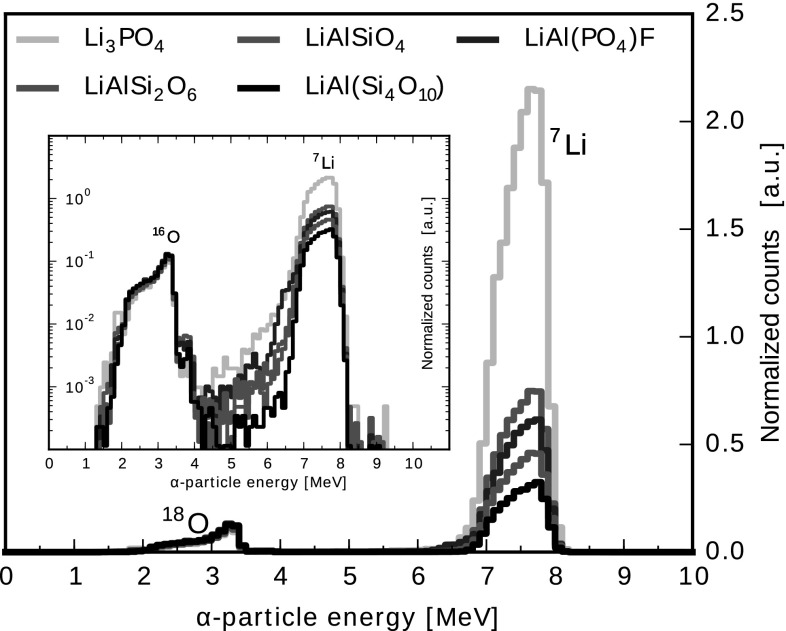
Normalized energy spectra of the α-particles produced in the reaction of protons at 852 keV with lithium atoms within the Li-mineral samples. The inset is a semi-log version of the spectra

The α-particle energy spectra presented in Fig. 2 were acquired during the irradiation of Li-mineral samples with protons with the energy 852 keV. Each spectrum exhibits α-particle distributions around 3 MeV and 7 MeV. The peak around 3 MeV consists of α-particles originating from the reaction ^18^O(p,α)^15^N, so these distributions are labeled O. The distributions between 7 MeV and 8 MeV are related to Li from the studied reaction, so this distribution is labeled Li. As can be seen in Fig. 2, the intensity of the Li-peak is proportional to the lithium concentration in the samples.

**Fig. 3:**
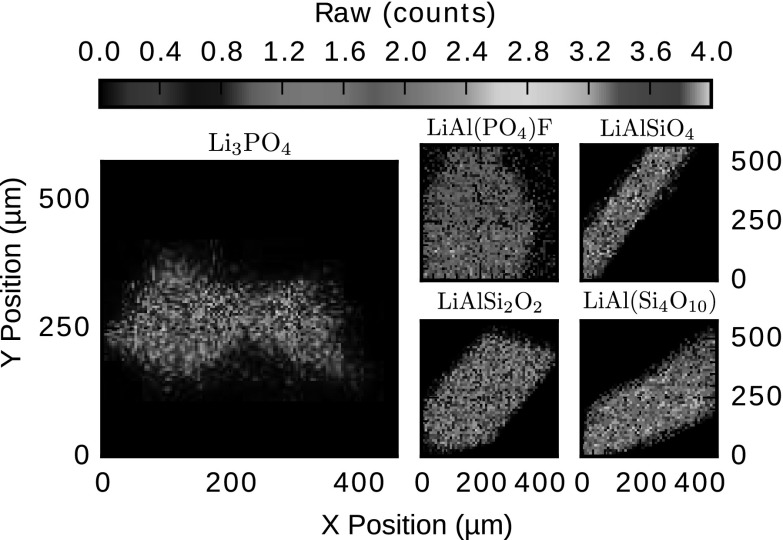
2D maps of the Li-minerals obtained with backscattered protons

**Fig. 4:**
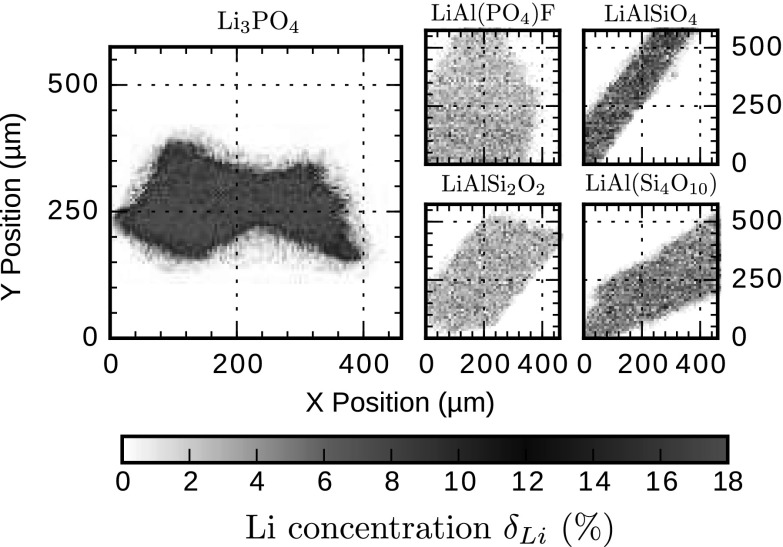
2D Li distribution maps of the Li-minerals

The analysis of a sample is performed in two steps. First, the elastically backscattered protons are measured in order to obtain a fast image of the sample, see Fig. 3. This 2D-preview helps us to select a desired scan area within a crystal. Thus, unnecessary scanning of areas outside the crystal is minimized, and so the time spent on analysis is optimized. The number of backscattered protons used for image reconstruction is called Raw counts. The Li measurement takes place in the second step of the analysis. A scanning area of interest is selected, within the boundaries of the image taken in the first step, and scanned with the proton beam, which causes protons and lithium to react. The α-particles emanating from lithium in the defined area are measured and used to create a 2D distribution map, see Fig. 4.

**Fig. 5:**
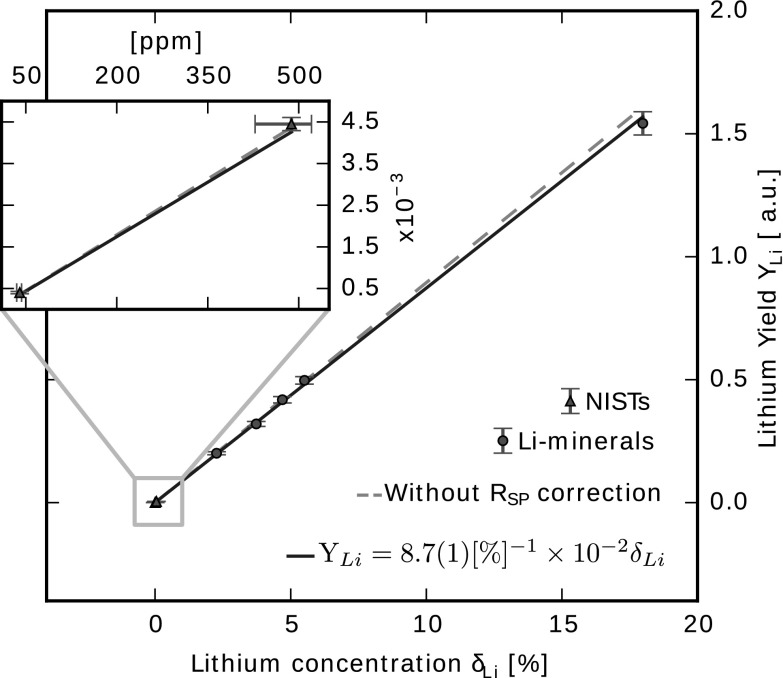
Lithium yield Y_Li_ as a function of lithium content in the crystals listed in Table 1. Based on Y_Li_ = R_SP_ C_Li_/Q, the Y_Li_ error bars were derived from error propagation calculations, taking into account that the R_SP_ range factor uncertainty is negligible, the C_Li_ uncertainty is calculated as $\sqrt {{\text{C}}_{\text{Li}} }$, and Q uncertainty is 3%

In Fig. 5, the correlation between measurements of different samples with different lithium concentrations δ_Li_ is shown; the δ_Li_ values are reported in wt-ppm. Lithium yield Y_Li_ exhibits a linear behavior with lithium concentrations δ_**Li**_ from 40 ppm to 18%. The correlation is given by the linear regression equation: δ_Li_ = 11.2(1)[%]Y_Li_, which was obtained using data from the Li-minerals. The NIST data fits very well with the linear regression as is shown in Table 1 as well as in an inset of Fig. 5. The continuous and dashed lines are a fit of the data with R_SP_ range corrections and a fit without R_SP_ range corrections respectively. At low lithium concentration, both fit lines can group the data. However, at high lithium concentration, the fit without R_SP_ range corrections does not pass through the last point.

**Table 1:** Lithium yield Y_Li_ and lithium concentrations δ_Li_ in Li-minerals and reference standard samples

Li-minerals	Y_Li_	Li concentration δ_Li_ (%)			
			Ranges (μm)	With R_SP_ corrections	Nominal values
		δ_Li_ = 11.18(10) Y_Li_	R_i_	δ_Li_ = 11.46(12) Y_Li_	δ_Li_
1. Lithiophosphate Li_3_PO_4_	1.54(5)	18.0(6)	27.4	17.7(6)	18.0
2. Eucryptite LiAlSiO_4_	0.50(1)	5.7(2)	28.6	5.7(2)	5.5
3. Amblygonite LiAl(PO_4_)F	0.42(1)	4.7(2)	27.9	4.8(2)	4.7
4. Spodumene LiAlSi_2_O_6_	0.32(1)	3.7(1)	27.7	3.7(1)	3.7
5. Petalite LiAl(Si_4_O_10_)	0.20(1)	2.3(1)	27.9	2.3(1)	2.3
NIST 610	4.4(2) × 10^−3^	497(20) ppm	–	501(20) ppm	$488_{ - 60}^{ + 33}$ ppm
NIST 612	4.0(3) × 10^−4^	45(3) ppm	–	46(3) ppm	$40_{ - 5}^{ + 3}$ ppm

δ_Li_ was obtained using the calibration shown in the Fig. 5. The δ_Li_ uncertainties are calculated using error propagation of the formulas obtained in the calibration. R_SP_ corrections were calculated based on the R_i_ ranges listed below. Nominal values of δ_Li_ for Li-minerals are concentrations calculated based on ideal stoichiometric formulas. In the case for NIST 610–612, nominal values of δ_Li_ were taken from the Ref [24, 25]

In a second analysis, eight tourmaline crystals were scanned using the same conditions as for the lithium concentration calibration. The boron concentrations in the tourmalines were determined using an equivalence between δ_B_ and Y_B_. It was acquired using the data from the NISTs and one tourmaline standard sample and this is summarized in Table 2.

**Table 2:** Boron yield Y_B_ and boron concentrations δ_B_ for standard reference samples

Crystal	Y_B_	B concentration δ_B_	
		δ_B_ = 1.98(08) Y_B_	Nominal values
Tourmaline standard	1.71(50)	3.40(20)%	3.27(1) % [27]
NIST 610	1.66(5) × 10^−2^	328(20) ppm	351 ppm [24]
NIST 612	1.74(7) × 10^−3^	34(2) ppm	32 ppm [25]

The concentrations shown in the column Nominal values are taken from the respective references

**Fig. 6:**
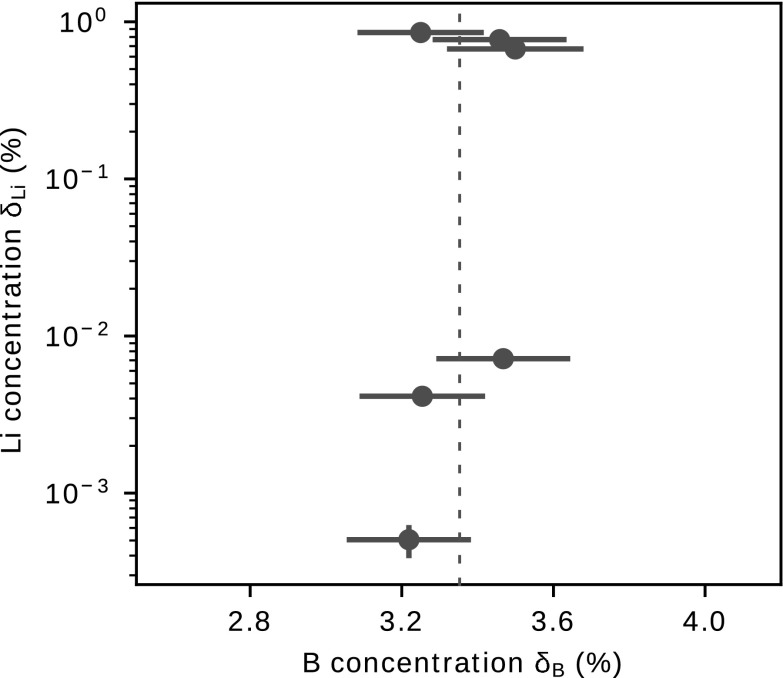
The lithium content correlation to boron content in the tourmaline samples. The dotted line indicates a boron concentration of 3.27%

To calculate the lithium content, the expression obtained previously, δ_Li_ = 11.5(1)[%] Y_Li_ was applied to the yields measured in the tourmalines. Figure 6 presents the concentrations of lithium and boron measured in the tourmaline samples. Boron concentration values are about 3.27%, as expected for tourmalines [15].

Regarding the lithium contents in the tourmaline samples, three sets of data present a significant lithium concentration around 0.7%. The remaining sets of data present low lithium contents, ranging from 10 ppm to 100 ppm. Tourmalines with lithium content are characteristic of the elbaite tourmaline group; for more details see Ref. [30] sample 61 Vbh and Ref. [31]. According to [32, 33], a very low concentration of Li in tourmaline is usually assumed to be insignificant, but tourmalines low in Li can present around 10–80 ppm of lithium content [31].

**Fig. 7:**
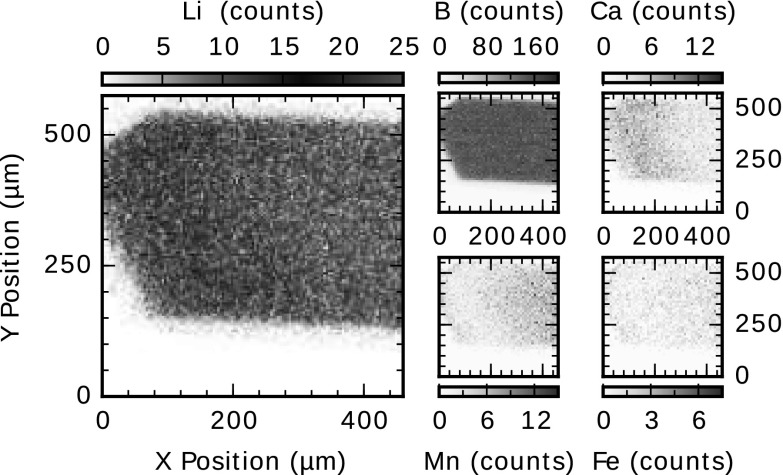
Lithium, boron, calcium, manganese and iron 2D maps in a tourmaline sample

Detection of heavy elements such as calcium and iron were possible to perform using the PIXE technique. As an example, Fig. 7 illustrates 2D map distributions of Ca, Mn and Fe in a tourmaline. Also the Li and B distributions are shown in the figure. Clear zonation patterns of Ca and Mn are distinguishable in the 2D maps. A continuous core-to-rim zoning where Ca increases as Mn decreases appears in the 2D maps of Ca and Mn presented in Fig. 7. The lithium 2D map also shows a continuous core-to-rim zoning. On the other hand, a homogeneous composition is exhibited in the 2D map of boron.

To investigate the detection limit of lithium, a lithium-free (below 0.001 ppm) Suprasil quartz crystal [26] was analyzed using a beam current of 2 nA for 2 h, and no lithium-related signal was found. To determine the sensitivity in this case, where the experiment has not resulted in any events, Poisson statistics for small signals was applied [34]. Thus, for a zero background spectrum, a sensitivity of 4.74 events or counts is given for a confidence level of 99% (3σ). Assuming a beam current of 4 nA for 1 h, the charge compensated by the live time of the system would be 5 × 10^5^, what leads a lithium yield value of 4.74/5 × 10^5^. From the calibration, this lithium yield should correspond to a concentration of δ_Li_ = 11.5 (4.75/5 × 10^5^)[%] = 1 × 10^−4^ [%], (1 ppm).

## Summary and conclusions

Using the reaction ^7^Li(p,α) ^4^He with proton energies at 852 keV, a fast and background free measurement of isolated α-peaks in standard and geological samples was obtained. The analytical technique presented in this work allowed acquiring lithium measurements from 4.3(8) ppm (a tourmaline sample) to 17.7(6)  % (lithiophosphate). Analysis of a Suprasil quartz crystal does not present any signal of lithium, which indicates that for 1 h analytical time at 4 nA, the detection limit of lithium is 1 ppm.

The presence and distribution of heavy elements such as iron, calcium and manganese in the lithium minerals or in the tourmaline samples were possible to measure using the PIXE technique simultaneously with the NRA. The results from tourmaline samples exhibit chemical zonations, which have being studied in Ref [16]. The compositional zoning within a specimen shows that an analytical investigation of an arbitrary section of a crystal can introduce possible errors. Hence, the technique performed in this work is a suitable method to track major chemical composition variations in a sample.

## Supplementary information

This section will not appear in the printed version of your paper but it will contain a link; the webpage containing the electronic supplementary information will appear when one clicks on the hyperlink. Here you can list the details of your research which would be too long for the main text, *e.g.* a larger number of spectra etc. Start with 1 for Figure and Table numbers in this section.
